# Evaluation of the implementation of a community health worker-led COVID-19 contact tracing intervention in Chiapas, Mexico, from March 2020 to December 2021

**DOI:** 10.1186/s12913-024-10590-3

**Published:** 2024-01-18

**Authors:** Zeus Aranda, Sandra Vázquez, Anuraag Gopaluni, Laura Martínez, Mayra Ramírez, Ariwame Jiménez, Daniel Bernal, Ana L. Rodríguez, Selene Chacón, Bruno Vargas, Isabel R. Fulcher, Dale A. Barnhart, The Partners In Health Cross-Site COVID-19 Cohort Technical Working Group

**Affiliations:** 1 Partners In Health Mexico (Compañeros En Salud), Compañeros En Salud AC, Calle Primera Pte. Sur 25, Colonia Centro, Ángel Albino Corzo, 30370 Chiapas México; 2 Departamento de Salud, El Colegio de La Frontera Sur, San Cristóbal de Las Casas, Chiapas, México; 3 Department of Biostatistics, Harvard T.H. Chan School of Public Health, Boston, MA USA; 4 Partners In Health Peru (Socios En Salud), Lima, Perú; 5 Escuela de Gobierno y Transformación Pública, Instituto Tecnológico de Monterrey, Ciudad de Mexico, México; 6 Instituto Nacional de Salud Pública/Escuela de Salud Pública de México, Cuernavaca, Morelos México; 7 Department of Global Health and Social Medicine, Harvard Medical School, Boston, MA USA; 8 Harvard Data Science Initiative, Boston, MA USA; 9 Partners In Health Rwanda (Inshuti Mu Buzima), Kigali, Rwanda

**Keywords:** Contact tracing, Community health workers, COVID-19, Mexico

## Abstract

**Background:**

Mexico is one of the countries with the greatest excess death due to COVID-19. Chiapas, the poorest state in the country, has been particularly affected. Faced with an exacerbated shortage of health professionals, medical supplies, and infrastructure to respond to the pandemic, the non-governmental organization Compañeros En Salud (CES) implemented a COVID-19 infection prevention and control program to limit the impact of the pandemic in the region. We evaluated CES’s implementation of a community health worker (CHW)-led contact tracing intervention in eight rural communities in Chiapas.

**Methods:**

Our retrospective observational study used operational data collected during the contract tracing intervention from March 2020 to December 2021. We evaluated three outcomes: contact tracing coverage, defined as the proportion of named contacts that were located by CHWs, successful completion of contact tracing, and incidence of suspected COVID-19 among contacts. We described how these outcomes changed over time as the intervention evolved. In addition, we assessed associations between these three main outcomes and demographic characteristics of contacts and intervention period (pre vs. post March 2021) using univariate and multivariate logistic regression.

**Results:**

From a roster of 2,177 named contacts, 1,187 (54.5%) received at least one home visit by a CHW and 560 (25.7%) had successful completion of contact tracing according to intervention guidelines. Of 560 contacts with complete contact tracing, 93 (16.6%) became suspected COVID-19 cases. We observed significant associations between sex and coverage (*p* = 0.006), sex and complete contact tracing (*p* = 0.049), community of residence and both coverage and complete contact tracing (*p* < 0.001), and intervention period and both coverage and complete contact tracing (*p* < 0.001).

**Conclusions:**

Our analysis highlights the promises and the challenges of implementing CHW-led COVID-19 contact tracing programs. To optimize implementation, we recommend using digital tools for data collection with a human-centered design, conducting regular data quality assessments, providing CHWs with sufficient technical knowledge of the data collection system, supervising CHWs to ensure contact tracing guidelines are followed, involving communities in the design and implementation of the intervention, and addressing community member needs and concerns surrounding stigmatization arising from lack of privacy.

**Supplementary Information:**

The online version contains supplementary material available at 10.1186/s12913-024-10590-3.

## Introduction

Between February 2020 and mid-September 2023, Mexico reported nearly 7.67 million confirmed COVID-19 cases and 334,506 deaths [1]. Mexico is the fifth country in the world with the highest number of confirmed COVID-19 deaths and the second in the Latina America and the Caribbean (LAC) region, after Brazil [1]. Within Mexico, the burden of COVID-19 disease has been unequally distributed, with people living in already marginalized areas being more likely to present with severe symptoms of COVID-19 disease [2]. Further, health care professionals in the country were particularly stricken by the pandemic, accounting for 8% of COVID-19 cases and 2% of deaths [3].

Chiapas, the poorest state in Mexico, has suffered disproportionately from the ongoing health emergency [4]. Hospitals in the region have been overwhelmed by the health care needs brought on by the pandemic, including an exacerbation of the pre-existing shortage of health professionals [5], medical supplies [6], and beds [7]. In this context, SARS-CoV-2 infection prevention and control interventions that can reduce the number of cases, especially among high-risk patients who are more likely to require hospitalization, are critically important. Contact tracing is one prominent SARS-CoV-2 infection prevention and control measure that may effectively reduce the number of new SARS-CoV-2 infections and the number of COVID-19-related deaths [8–10].

In March 2020, Compañeros En Salud (CES), the non-governmental sister organization to Partners In Health (PIH) in Mexico, implemented a contact tracing intervention for eight rural communities in the Fraylesca and Sierra regions of Chiapas. The intervention was designed according to international recommendations [11–13] and drew on experiences from other PIH sites [14–16]. Considering CES’s scarce human and material resources and limited telecommunication access in most of the rural communities served by the organization, the intervention was implemented using CES's community health worker (CHW) workforce. CHWs have supported similar contact tracing interventions for the control of other infectious diseases, such as Ebola in Congo, Sierra Leone, Guinea, and Liberia; tuberculosis in Kenya, Peru and Spain; and HIV in Haiti, Malawi, Uganda, and South Africa [15–23].

Contact tracing by CHWs for COVID-19 has also been reported in other settings, including by PIH-led teams in the United States (US) and Haiti [16, 24]; a public–private intervention in New York (US) [25]; government-led interventions in Bangladesh, India, Nepal, Thailand, and Vietnam [26, 27]; government-led interventions in Oman, Nigeria, Uganda, Rwanda, and South Africa [28, 29]; and programs supported by the Partnership to Accelerate COVID-19 Testing (PACT) in some African Union countries [30]. However, the scientific literature evaluating the implementation of these CHW-led contact tracing for COVID-19 remains scarce.

In May 2023, the World Health Organization (WHO) lifted the Public Health Emergency of International Concern (PHEIC) for COVID-19 [31]. However, the WHO stressed the importance of State Parties continuing to conduct research on COVID-19 as a pillar of preparedness for future disease outbreaks [32]. Future epidemics may arise from SARS-CoV-2 variants capable of evading established immunity from vaccines and previous infections [33] and other known or unknown pathogens [34]. Moreover, it has been estimated that the annual probability of occurrence of extreme epidemics can increase in the coming decades, as a consequence of an increased emergence of diseases from zoonotic reservoirs associated with environmental change [35].

In line with the WHO call for research on COVID-19, we conducted an evaluation of our CHW-led contact tracing intervention for COVID-19, including a detailed description of its implementation, the assessment of key process indicators over time and their association with demographic characteristics of contacts, as well as a detailed analysis of factors that may have hindered implementation of our intervention. To our knowledge, this study represents the first evaluation of a CHW-led contact tracing intervention for COVID-19 in the LAC region. Our study will serve as a reference for decision-makers working in disease outbreak control, especially those working in underserved areas, as it underscores some key aspects that may jeopardize the success of CHW-led in person contact tracing interventions, such as training and supervision of CHWs, data collection systems and quality, and communities’ acceptability of the intervention. Hopefully, our study will help prevent the omission of these important aspects from the intervention design stage, which may have a positive impact on the outcomes of such interventions if implemented in future disease outbreaks.

## Methods

### Study setting

Compañeros En Salud (CES) supports ten rural public outpatient clinics in ten communities in the Fraylesca and Sierra regions of Chiapas with a total population of 11,707 inhabitants [36]. This rural area has extremely limited telecommunication and internet access. CES’s regular support activities include financial support, training, and supervision for health professionals working in clinics in addition to delivery of medical supplies. Of the ten communities supported by CES, eight accepted the CHW-led contact tracing intervention, which was shared and discussed with local community leaders prior to implementation through a series of meetings. The reason why two communities did not accept the intervention was mainly due to mistrust issues. At the beginning of the COVID-19 pandemic, CES engaged a workforce of 45 CHWs for COVID-19 contact tracing. These CHWs had previously acted as a bridge between patients and the clinics and provided biosocial accompaniment via home visits to persons with chronic diseases (predominately with diabetes and hypertension) and pregnant and postpartum women. CHWs who participated in contact tracing were incentivized with an increase in their stipend from 600 to 1,400 Mexican Pesos [MXN] (approximately 30 to 70 USD).

### Evolution of community contact tracing for COVID-19

**Fig. 1:**
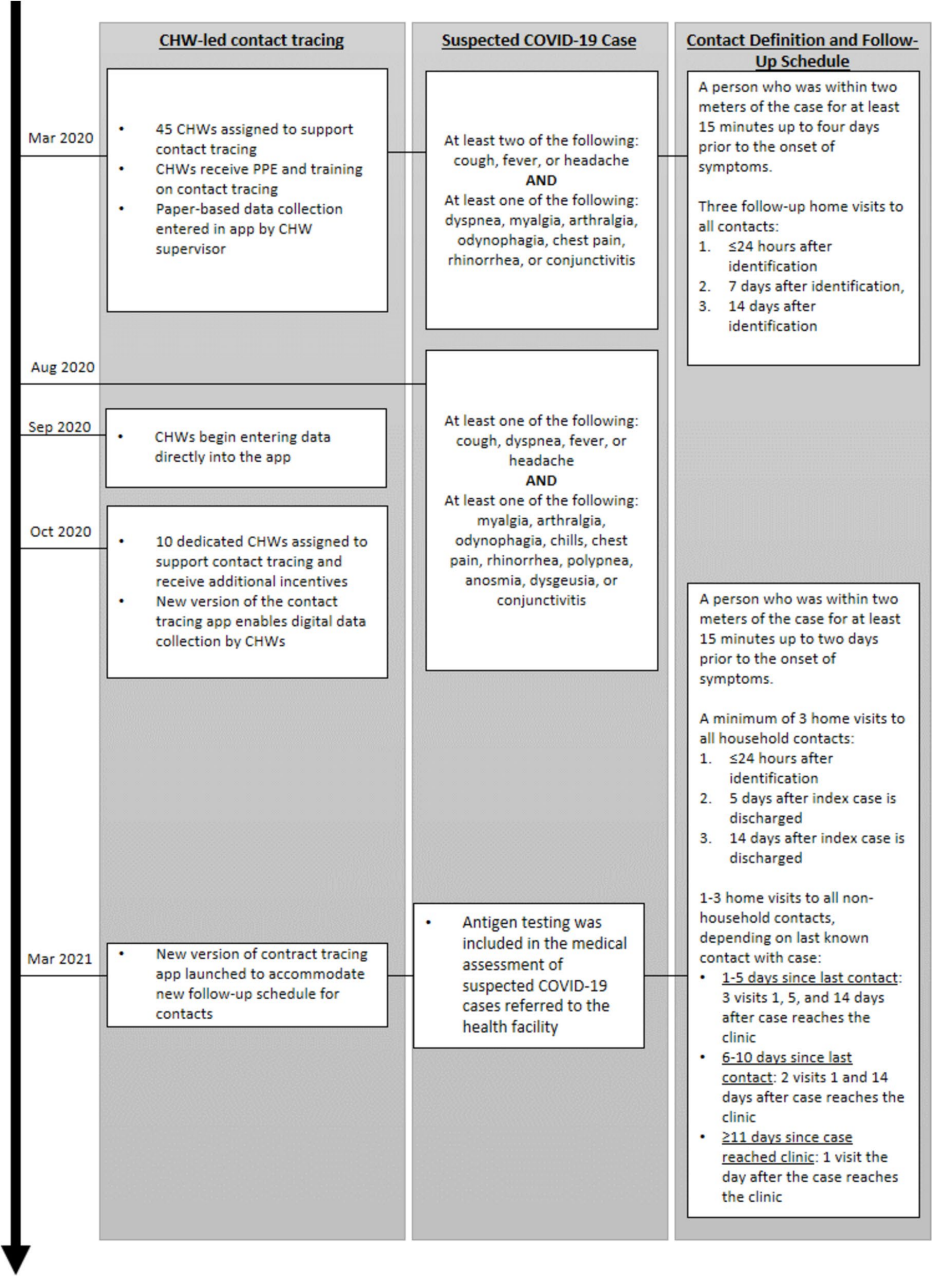
Timeline of the evolving Compañeros En Salud community contact tracing intervention. CHW: community health worker

CHW-led contact tracing was implemented for all patients who accepted it voluntarily from the eight outpatient clinics who fulfilled the Mexican Ministry of Health’s (MoH) definition for a suspected COVID-19 case in March 2020 (Fig. 1). As a result of the uncertainties we faced and despite the unavailability of COVID-19 tests, which are the starting point for most contact tracing initiatives, we went ahead with ours and started following up suspected symptomatic cases and their contacts, based on the precautionary principle, founded on the idea that uncertainty is not sufficient justification for not taking measures to prevent the occurrence of an adverse outcome [37]. Nursing assistants, nurses, and doctors at the clinics filled out the MoH’s official case notification form and listed a roster of contacts for each COVID-19 “index case” on a paper-based form. The definition of “contact” aligned with international and national guidelines [11], and was operationalized by health professionals at the clinics by asking the index case to list anyone who a) lived in the same household or with whom the case has had close contact, such as family; b) was in the same enclosed space such as a road trip, church or other public gathering, listing only the people he/she was seated next to; or c) was within touching distance for a period longer than a greeting for up to four days prior to the onset of the symptoms. Each day, contact rosters were given to the CHW facilitator in each community for distribution to CHWs based on their assigned area. CHWs followed up the contacts at their home. To ensure the safety of CHWs, CES provided them with personal protective equipment (PPE), including face shields, face masks and hand sanitizer, and instructed them on communication skills to help them avoid stigmatization of cases and contacts as well as preserve their privacy, and on the SARS-CoV-2 infection prevention and control protocol, which entails interacting with cases and contacts in an outdoor setting with at least six feet of distance. In patients showing certain symptoms pointing to a more severe COVID-19 state, CHWs were instructed on how to use medications such as acetaminophen, oral rehydration salts, and an oximeter.

During the home visit, CHWs collected basic demographic data on all named contacts and screened them for COVID-19 compatible symptoms. If the contact presented with symptoms that were compatible with a definition of a suspected COVID-19 case during the follow-up visits, such as shortness of breath, cyanosis, tachypnea or chest pain, they were referred to the closest clinic for medical assessment and received CHW home visits as a COVID-19 case. In addition to screening for COVID-19-related symptoms, CHWs educated contacts on the importance of quarantine and adoption of additional hygiene measures; provided instructions on steps to take if COVID-like symptoms developed; identified contacts who were at risk of severe COVID-19, including persons 60 years of age or older, pregnant women, persons who are obese or overweight, and those suffering from immunosuppressive, chronic, cardiac, pulmonary, renal, hepatic, blood, or metabolic diseases, and educated them on the importance of increased caution [38]; assessed socioeconomic needs of participants; and provided socioeconomic support, such as food packages and hygiene kits, as necessary. At first, due to connectivity issues, CHWs recorded information on demographics and symptoms for each contact and visit on paper-based forms, which were later entered into an electronic CommCare app (Dimagi, Inc., 2022) by CHW supervisors and the CES monitoring and evaluation team.

Throughout the pandemic, we made several adaptations to improve the delivery of the contact tracing program (Fig. 1). Following guidance from the national government, the definition of a suspected case was revised in August 2020. In September 2020, CHWs were trained to enter data directly into the CommCare app offline during home visits and to upload the information to the online server daily upon returning to the clinic. In October 2020, contact tracing responsibilities were transferred from the 45 CHWs to a workforce of ten specialized CHW with the rest of the initial contact tracing cadre returning to their original roles. This, due to the need to continue to provide home follow-up for chronically ill patients—including the provision of medications—and pregnant and puerperal women, and to promote the use of CES-supported health facilities among these populations. A major concern of CES was the detection of a decline in utilization of health care services during the summer of 2020 [39], so one of the strategies to regain pre-pandemic utilization levels was communication by CHWs of the safety measures that had been implemented at CES-supported facilities to protect patients from SARS-CoV-2 infection.

The CHWs specialized in contact tracing for COVID-19 were CHWs or auxiliary nurses working in the communities who expressed an interest to carry out COVID-19-related tasks. They received additional training and materials, including electronic devices for data entry and PPE, an increased remuneration from 1,400 to 4,000 MXN for CHWs (approximately 70 to 200 USD) and from 2,000 to 4,000 MXN (100 to 200 USD) for auxiliary nurses, and were formally hired by CES as full-time staff, which allowed for the provision of social security benefits. Finally, in March 2021, antigen testing was included in the medical assessment of suspected COVID-19 cases referred to the health facility to confirm their diagnosis, changes were made to the definition of contacts and the contact tracing follow-up schedule, and these changes were integrated into an updated version of the CommCare app.

### Study population and data sources

We extracted data on the index cases, contacts, and contact tracing visits from all three versions of the contact tracing App for those individuals who had given informed consent to participate in the study (or their legal guardians if the individual was younger than 16 years of age). After removing identifiable information and harmonizing variables across the three versions of the App databases, we created a combined dataset of all named contacts and basic demographic information for each contact as given by the index case. Similarly, we created a combined dataset of information collected by CHWs during contact tracing visits. Finally, we merged the combined contact roster and contact tracing visit datasets by contact ID. Our final dataset included 2,177 contacts named by index cases at the eight CES-supported clinics from the start of the program in March 2020 through November 2021, and information collected during 2,894 total visits for 1,187 unique contacts who were located by CHWs during this period.

### Definition of outcomes

We evaluated three outcomes: contact tracing coverage, successful completion of contact tracing, and incidence of suspected COVID-19 among contacts. Contact tracing coverage was defined as whether a named contact received at least one in-person contact tracing visit. The definition of successful completion of contact tracing varied according to the guidelines in place at the time (Fig. 1). Between March 2020 and March 2021, the criterion for successful completion of contact tracing was either a) the contact was followed up until they became a suspected case, or b) the contact received at least three total contact tracing visits. After March 2021, the criterion for successful completion of contact tracing was a) the contact was followed up until they became a suspected case; or b) the contact lived in the same household as the case and received at least two additional visits after the case was discharged; or c) the contact did not live in the same household as the case and had at least one follow-up visit at least 14 days after the case reached the clinic. Our definition of successful completion of contact tracing after March 2021 differs slightly from the criteria presented in Fig. 1 due to a lack of data on the date of contacts’ last encounter with the case in our dataset. Our third outcome was incidence of suspected COVID-19 diagnosis, which was based on symptoms reported by the contact with the exact definition varying over time according to Fig. 1.

### Statistical analysis

We created a cascade of care for community contacts of suspected and confirmed COVID-19 cases by reporting the numbers of contacts who were identified, achieved contact tracing coverage and successful completion of contact tracing, and developed suspected COVID-19. We reported the probability of successfully completing each stage in the cascade conditional on completing the previous stage. We repeated this process for each month of the contact tracing program and plotted each of these indicators over time.

For all named contacts, we calculated frequencies and percentages of demographic characteristics including sex, age, time period, and community. Time period was defined as before or after the definition of contacts and contact tracing schedule changed in March 2021. For interviewed contacts, we additionally reported comorbidities and whether the contact was from the same household as the case. Comorbidities were self-reported by interviewed contacts. We compared the prevalence of diagnosed hypertension and diabetes and tobacco use to population-level estimates from the state of Chiapas to investigate possibility of misclassification. To measure the association between contact tracing coverage and sex, age, time period, and community we used univariate logistic regressions with cluster–robust standard errors to adjust for clustering of contacts by case. Among the subset of individuals who successfully initiated contact tracing, we assessed the association between successful completion of contact tracing and sex, age, time period, community, whether or not the contact had any comorbidities, and whether or not the contact was part of the same household as a case again using univariate logistic regressions with cluster–robust standard errors. Because age and sex are common determinates of many comorbidities, we also assessed for whether there was an association between successful completion of contract tracing and having at least one comorbidity after adjusting for the potential confounders of age and sex. Similarly, we also assessed whether successful completion of contract tracing was associated with being a household contact after adjusting for age and sex, as well as after adjusting for time period, because being a household contact impacted the definition of successful completion of contact tracing during the post-March 2021 time period. Finally, we assessed the association between having a suspected COVID-19 diagnosis and demographic characteristics among a subset of individuals who successfully completed contract tracing using the same univariate and multivariate logistic regression models described for successful completion of contract tracing. We used the 0.05 level of significance for all tests of association and reported two-sided *p*-values. We implemented all analyses using Stata version 17.

## Results

### Cascade of care for contacts of COVID-19 cases

**Fig. 2:**
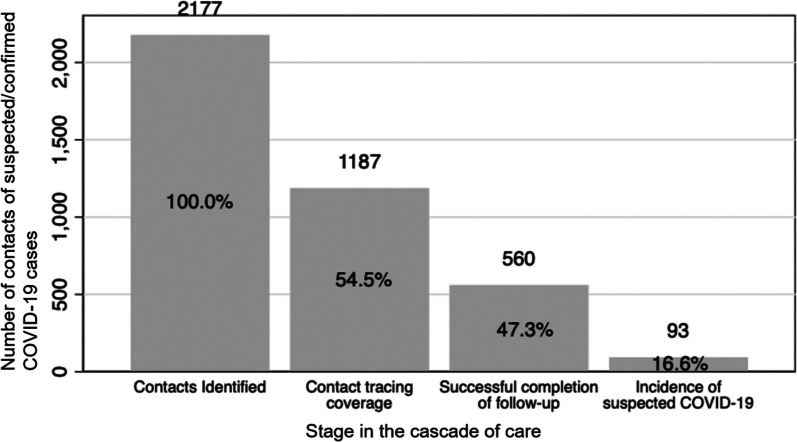
Cascade of care for community contacts of suspected/confirmed COVID-19 cases at Compañeros En Salud

**Fig. 3:**
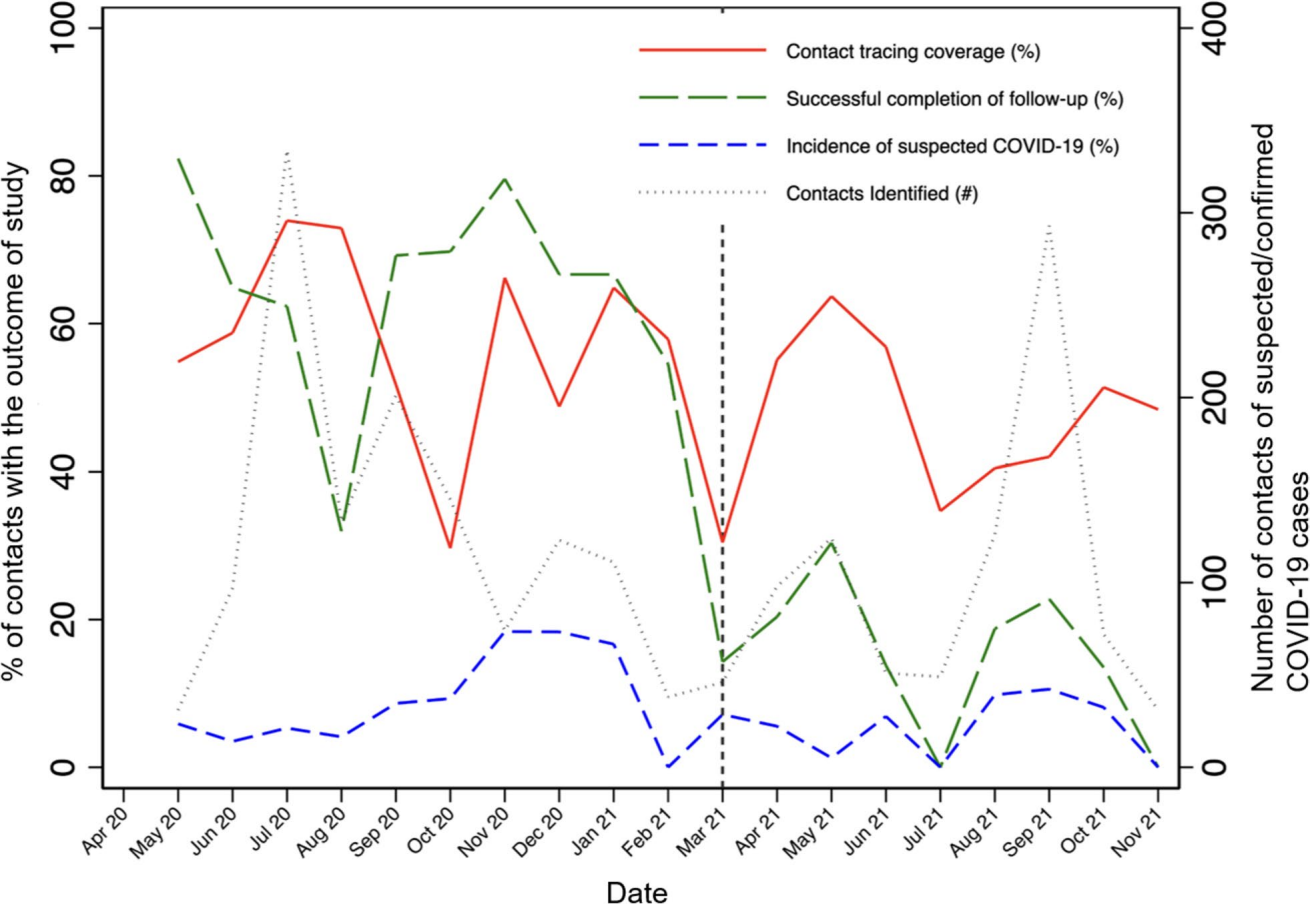
Evolution of the Compañeros En Salud-contact tracing intervention outcomes from April 2020 to November 2021. Monthly percentage of contact tracing coverage, successful completion of contact tracing, and contacts who became suspected COVID-19 cases, accompanied by the number of contacts identified in the supported rural communities

Of the 2,177 contacts who were identified by suspected or confirmed COVID-19 cases, 54.5% were home visited at least once by CHWs and nearly half (47.3%) of contacts who received at least one home visit successfully completed contact tracing (Fig. 2). CHWs reported in the system refusal of contact tracing during the initial approach by 73 individuals (3.4% of all identified contacts). Among contacts who successfully completed contact tracing and for whom symptomatic information was available (*n* = 560), 16.6% met the criteria for becoming a suspected COVID-19 case. When assessing how these outcomes changed over time, we observed two periods, June 2020 to October 2020 and August 2021 to October 2021 with spikes in the number of contacts reported (Fig. 3). We also observed a notable reduction in the proportion of contacts who successfully completed contact tracing before and after the implementation of new contact tracing guidelines in March 2021 (62.1% vs. 20%).

### Predictors of contact tracing coverage

We observed that contact tracing coverage was significantly higher among male contacts compared to female contacts (57.5% vs. 51.8%, *p* = 0.006) and before the change in the contact tracing algorithm in March 2021 compared to after (58.5% vs. 48.4%, *p* < 0.001). Contact tracing coverage also varied between communities (*p* < 0.001). Age was not associated with differences in contact tracing coverage (Table 1).

**Table 1:** Association between contact tracing coverage (initial contact) and demographic characteristics (*n* = 2177)

**Characteristic**^b^	**Frequency (N)**	**Contact tracing coverage (%)**	***p*-value**
**Sex**			0.006^+^
Male	1058	57.47	
Female	1117	51.75	
*Missing*	*2*	*50.00*	
**Age (years, categorized)**			0.921
0–18	703	62.02	
19–39	606	61.39	
40–49	184	59.24	
50–59	113	60.18	
60–69	68	64.71	
> 70	43	65.12	
*Missing*^a^	*460*	*28.26*	
**Community**			< 0.001^+^
C1	32	43.75	
C2	576	44.44	
C3	81	80.25	
C5	184	78.80	
C7	154	81.17	
C8	167	51.50	
C9	70	42.86	
C10	913	51.04	
*Missing*^a^	0	0.00	
**Period**			< 0.001^+^
*March 2020-Feb 2021*	*1,312*	*58.54*	
*After March 2021*	*865*	*48.44*	
*Missing*^a^	*0*	*0.00*	

^+^*p*-value < 0.05

^a^Missing values are excluded from statistical testing

^b^Demographic information on non-contacted individuals was reported by the root suspected COVID-19 case

### Demographic characteristics of located contacts

Of the 1,187 contacts who received at least one contact tracing visit, about half were men (51.3% men vs. 48.7% women; Table 2), most were under 40 years old (76.4%), and most lived in the same household as the case (88.1%). Of the 14.1% of individuals with at least one comorbidity, diabetes (24 cases among all contact), hypertension (18 cases among all contacts), and pulmonary disease (12 cases among all contacts) were the most common. Table 2 provides a summary of the demographic characteristics of the contacts followed up by the program.

**Table 2:** Demographics among interviewed contacts (*n* = 1187)

Characteristic	Frequency (N)	Percent (%)
**Sex**		
Male	608	51.26
Female	578	48.74
*Missing*^a^	*1*	*0.08*
**Age (years, categorized)**		
0–18	436	41.25
19–39	372	35.19
40–49	109	10.31
50–59	68	6.43
60–69	44	4.16
> 70	28	2.65
*Missing*^a^	*130*	*10.95*
**Community**		
C1	14	1.18
C2	256	21.57
C3	65	5.48
C4	145	12.22
C5	125	10.53
C6	86	7.25
C7	30	2.53
C8	466	39.26
*Missing*^a^	*0*	*0.00*
**Period**		
*March 2020-Feb 2021*	768	64.70
*After March 2021*	419	35.30
*Missing*^a^	*0*	*0.00*
**Contact from same household as case**		
Yes	645	88.11
No	87	11.89
*Missing*^a^	*455*	*38.33*
**Any comorbidity**		
Yes	104	14.11
No	633	85.89
*Missing*^a^	*450*	*37.91*
**Comorbidity**^b^		
Diabetes (population ≥ 20 y.o.)	23 (477)	5.15 (7.80 in Chiapas^c^)
Hypertension (population ≥ 20 y.o.)	17 (440)	3.86 (16.2 in Chiapas^c^)
Lung Disease	12 (712)	1.69
Obesity	8 (710)	1.13
Smoking Status (population 12–65 y.o.)	6 (448)	1.34 (7.60 in Chiapas^d^)
Pregnancy (women of childbearing age, 15–49 y.o.)	3 (329)	0.91
Heart Disease	4 (696)	0.57
Kidney Disease	4 (709)	0.56
Stroke	4 (709)	0.56
Liver Disease	2 (709)	0.28
Cancer	0 (709)	0.00
Malnutrition	0 (694)	0.00

^a^Missing values are excluded from calculation of proportions

^b^For the general population unless otherwise specified

^c^Data from the National Health and Nutrition Survey conducted by the National Institute of Public Health in Mexico (INSP) (2018) [40]

^d^Data from the National Survey on Drug, Alcohol and Tobacco Use conducted by the INSP (2016) [41]

Sex was significantly associated with successful completion of contact tracing, with males having a lower completion rate than females (44.7% vs. 50%, *p* = 0.049). Community of residence of the contact (*p* < 0.001), and the implementation period were also associated with successful completion of contact tracing, with higher completion rate before March 2021 than after (62.1% vs. 20%, *p* < 0.001). However, there were no significant associations between successful completion of contract tracing and age, having at least one comorbidity, or being a household contact. This lack of association between successful completion of contract tracing and having at least one comorbidity persisted after adjusting for age and sex (*p* = 0.331). Similarly, the lack of association between successful completion of contract tracing being a household contact persisted after adjusting for age and sex (*p* = 0.162) or when adjusting for time period (*p* = 0.146) (Table 3).

**Table 3:** Association between successful completion of contact tracing and characteristics (*n* = 1184)

**Characteristic**	**Frequency (N)**	**Successful Completion Percentage (%)**	***p*-value**
**Sex**			0.049^+^
Male	607	44.65	
Female	576	50.00	
*Missing*^a^	*1*	*100.00*	
**Age (years, categorized)**			0.968
0–18	433	48.50	
19–39	372	47.31	
40–49	109	50.46	
50–59	68	47.06	
60–69	44	45.45	
> 70	28	50.00	
*Missing*^a^	*130*	*40.77*	
**Community**			< 0.001^+^
C1	14	78.57	
C2	256	55.08	
C3	62	51.61	
C4	145	46.21	
C5	125	49.60	
C6	86	31.40	
C7	30	30.00	
C8	466	45.28	
*Missing*^a^	*0*		
**Period**			< 0.001^+^
*March 2020-Feb 2021*	768	62.11	
*After March 2021*	416	19.95	
*Missing*^a^	*0*		
**Any Comorbidity**			0.169
Yes	104	57.69	
No	633	65.09	
*Missing*^a^	*447*	*19.69*	
**Contact from same household as case**			0.374
Yes	645	39.22	
No	84	51.19	
*Missing*^a^	*455*	*58.02*	

^+^*p*-value < 0.05

^a^Missing values are excluded from statistical testing

### Incidence of suspected COVID-19 among contacts

There was no association between becoming a suspected COVID-19 case and sex, age, having any comorbidity (even when adjusting for age and sex, *p* = 0.253), or being a household contact (even when adjusting for age and sex, *p* = 0.284, or when adjusting for time period, *p* = 0.083) (Table 4).

**Table 4:** Association between suspected COVID diagnosis and key characteristics including comorbidities (*n* = 560)

Characteristic	Frequency (N)	Percentage (%) with suspected COVID diagnosis	*p*-value
**Sex**			0.337
Male	271	15.13	
Female	288	18.06	
*Missing*^a^	*1*	*0.00*	
**Age (years, categorized)**			0.397
0–18	210	18.10	
19–39	176	16.48	
40–49	55	10.91	
50–59	32	12.50	
60–69	20	15.00	
> 70	14	21.43	
*Missing*^a^	*53*	*18.87*	
**Any Comorbidity**			0.416
Yes	60	20.00	
No	412	13.35	
*Missing*^a^	*88*	*29.55*	
**Contact from same household as case**			0.146
Yes	253	24.11	
No	43	11.63	
*Missing*^a^	*264*	*10.23*	

^+^*p*-value < 0.05

^a^Missing values are excluded from statistical testing

## Discussion

Analysis of this CHW-led contact tracing intervention allowed us to describe the work carried out by CHWs in the eight CES-supported rural communities in Chiapas, Mexico, and identify areas of improvement for the intervention. Over half of the contacts identified by suspected or confirmed COVID-19 cases received at least one home visit by CHWs and over a quarter successfully completed contact tracing according to intervention guidelines. Although we observed statistically significantly higher contact tracing coverage among males and significantly higher successful completion of contact tracing among females, in practice, contact tracing coverage and successful completion were less than 60% for both genders, pointing to a need for general programmatic strengthening. In our program, approximately one in six contacts with complete contact tracing became a suspected COVID-19 case, which is comparable to reports from Uganda (13%) and Nigeria (11%), and some regions of the United States (7.8%-28.9%), but higher than in Rwanda (2%) [29, 42]. However, comparability with these other studies is limited as our program experienced limited availability of COVID-19 tests and consequently considered COVID-19-like symptoms for identification of index cases and incident COVID-19 among contacts while the above studies used diagnostic tests from the start to diagnose COVID-19 among index cases, contacts, or both. In the case of Oman, whose national CHW-led contact tracing program also considered COVID-19 suspicion, the figure was substantially higher than ours, at 45% [28]. Furthermore, in our study we did not find an association between age and becoming a suspected COVID-19 case, which contrasts with the findings of other studies, according to which a lower incidence of suspected COVID-19 cases would be expected among younger contacts. Several studies have found a lower susceptibility to SARS-CoV-2 infection among younger contacts of COVID-19 cases [43, 44], as well as a lower likelihood of developing symptoms among younger contacts infected with the virus [45]. However, our results regarding COVID-19 incidence among contacts should be viewed with caution, as we used suspected COVID-19 cases as a proxy indicator of COVID-19 cases and the algorithm used to identify suspected COVID-19, provided by the Mexican MoH, could not be validated with the participant population due to the lack of COVID-19 testing in the study setting.

The performance of our contact tracing program was poorer than what had previously been achieved by other contact tracing interventions for COVID-19 with participation of CHWs. In Nigeria (which included only CHWs) and Western Cape Province, South Africa (which included CHWs and volunteers), government-supported programs reached at least 90% of contacts, whereas in Uganda (which included CHWs, volunteers, students, and epidemiologists) and Rwanda (which included CHWs, volunteers, and students) over 89% of contacts successfully completed contact tracing [29]. The community engagement specialist workforce, implemented by a public–private partnership in New York, reached 71% of contacts [25], whereas the govenrment-led intervention in Oman obtained full coverage and follow-up of all contacts [28]. Other contact tracing programs that did not include CHWs, including interventions led by universities [46, 47] and health care institutions and health departments [42] in the US achieved more than 70% of contact notification. It is worth mentioning that unlike our intervention, which relied exclusively on in-person visits due to poor telecommunications coverage in the region, most of these contact tracing interventions were remote or combined remote and in-person activities.

### Implementation challenges and recommendations for practice

Although future research is needed to explore the reasons behind the relatively low numbers achieved by our intervention, there are a few possible explanations. First, because our program was implemented in a region with extremely limited telephone and internet connection, all contact tracing visits had to be conducted in person. In-person contact tracing is more time-consuming and dangerous than remote contact tracing, is less likely to occur during early morning and evening hours when people may be home, and offers fewer opportunities for repeated efforts to locate missing contacts. Second, from an intervention delivery point of view, lack of sufficient training and supervision of CHWs conducting contact tracing may have negatively affected the intervention outcomes. This intervention was being implemented at a time when the COVID-19 pandemic was placing a strain on health-facility based staff, limiting the number of external visitors local authorities would welcome into their community, and creating logistical barriers to CHWs attending in-person training. The general lack of telecommunications compounded these challenges by reducing opportunities to provide continuous remote training and supportive supervision. Consequently, when training sessions were able to be held the CHWs were at different stages of learning, and individual learning needs could not always be met due to human resource and time constraints. In addition, some CHWs reported a lack of supervision during their fieldwork, which led to unresolved doubts about the contact tracing algorithm and the use of the CommCare app, especially after CHWs switched from paper-based data collection to app-based data collection in September 2020 and after major changes to the program were incorporated in March 2021. Ultimately, this lack of support likely affected the quality of both the contact tracing program and the data collected. Furthermore, the reduction of CHWs working on contact tracing from 45 to 10 in October 2020 could have led to overloading of the remaining contact tracers, limiting the maximum achievable coverage and completion of contact tracing.

A third key challenge faced by our intervention was community engagement and acceptance of the contact tracing program. According to the data provided by the CHWs, only 3.4% of all identified contacts refused their home visits. However, we believe that this figure was likely to be higher, as it was noted that some CHWs, when the contact refused visits, did not specify this information in the CommCare app. As a result, in the analysis these contacts were not counted as contacts who refused the intervention, but rather as contacts who were never reached out by the CHWs. Contact refusal of the intervention was also identified in an assessment of risk perception, influences, knowledge, attitudes, and social stigma manifestations regarding COVID-19 conducted by CES in seven of the supported communities in December 2020. This assessment included surveys, semi-structured interviews, and focus group discussions with households, health care providers at health facilities, and CHWs. Interviewees identified knowledge gaps and misconceptions surrounding the CES SARS-CoV-2 infection prevention and control program among households, as well as villagers’ attitudes counterproductive to the control of the virus, such as refusing permission for CHWs to conduct home visits, for fear that confidentiality would be broken and that the rest of the community would learn of their health condition and ostracize them [48]. To address these concerns, CES implemented COVID-19 risk and anti-stigma communication campaigns in some of the communities starting at the end of 2020, with the hope of increasing community engagement in SARS-CoV-2 control and prevention activities, including contact tracing [49]. After the campaigns, clinic health staff and CHWs reported an increased willingness on the part of suspected COVID-19 cases and their contacts to receive home visits from CHWS [48]. CES is aware of the importance of addressing these concerns among the population and of placing the community at the center of the intervention to ensure the success of its implementation and will continue to strengthen facilitators (such as sense of collective responsibility, feeling of personal benefit, participation in the production of contact tracing systems, and perception of the intervention as useful) and combat barriers (such as mistrust, fear of stigmatization, and privacy concerns) for engagement with contact tracing among intervened communities [50]. In addition, routine monitoring and evaluation of progress towards community engagement will be key to identifying and overcoming failures in this process [51].

In our context of extremely limited telephone and internet connection, conducting data collection with digital tools posed substantial technical obstacles but also offered significant advantages over the paper-based system. For instance, connectivity limitations delayed the process through which clinic nurses could upload lists of newly identified contacts into the App, impeding CHWs’ ability to conduct contact tracing and enter home visit information into the App. Also, challenges in downloading the latest versions of the CommCare app, technical problems with the mobile devices used for data collection, and regular power outages in the communities sometimes prevented CHWs from recording their home visits. The design of the App could also have negatively impacted data collection because CHWs’ user experience was not prioritized during the design process. Using what is known as human-centered design, which entails observing the intended final user’s use of the tool, creating spaces for sharing user feedback on app design features, and conducting iterative pilot testing to incorporate observations and feedback ideas into the app [52], has been shown to be effective in improving visit coverage by CHWs [53]. Incorporating human-centered design principles during the development of the App may have improved CHWs ability to interact with the tool. Despite these challenges, using a digital tool offered many meaningful benefits. For instance, the skip patterns within the App could guide CHWs on actions to be taken in specific situations, such as indicating the date to arrange the next visit or what to do in case the contact presents COVID-like symptoms. It also limited in-person contact between CHWs and health care professionals in the clinics, potentially reducing exposure to SARS-CoV-2. Compared to paper-based data collection, which involved transferring paper-based data to a digital database for monitoring and evaluation, a real-time digital data collection system avoided duplication of work and reduced the possibility of some data entry errors. The use of the App also corrected other issues with paper forms, such as loss, tearing, and the possible spread of the virus through their surface, as well as enhanced patient privacy, since only users with credentials could access the App data. Due to the substantial benefits offered by digital-based contact tracing tools, we would encourage future CHW-led contract tracing efforts to overcome technical barriers to implementation by investing more in the initial design of the tools using feedback from the CHWs and by investing more resources in continuous monitoring of data quality throughout the contact tracing program’s lifespan.

All of the described challenges affecting the CES CHW-led contact tracing intervention, including those related to training, supervision, data collection systems, and community engagement, have been previously reported by other CHW interventions [28, 54, 55]. These elements have been also identified by the WHO as key elements for CHW program optimization [56]. A qualitative evaluation of a volunteer-led COVID-19 contact tracing intervention in the US found that contact tracers emphasized the value of receiving ongoing training and consistent, supportive supervision to ensure intervention success [57]. In the future, it will be crucial to work on all these aspects to improve implementation outcomes.

It was the first time a contact tracing intervention was implemented by CES. Moreover, the implementation took place in a health emergency context, characterized by a high degree of uncertainty and the need for a rapid response to contain the pandemic. At the time of intervention design in March 2020, evidence and guidelines on the use of contact tracing for COVID-19 were still very poor. However, CES drew on the experiences of other PIH sites using community-based contact tracing strategies to contain previous infectious disease outbreaks [14–16], as well as leveraged one of the NGO’s greatest assets—its CHW workforce—to design its own contact tracing intervention for COVID-19. Despite the barriers posed by the underserved environment where CES works, which explain the poor intervention outcomes, the organization decided to go ahead with implementation because it felt a moral obligation to initiate and sustain SARS-CoV-2 infection prevention and control strategies that could be beneficial to the population, even if the implementation conditions were not optimal. This reasoning was in line with PIH’s principles, reflected in the organization’s work in bringing any available prevention and treatment strategies for HIV/AIDS and multi-drug resistant tuberculosis to populations in underserved settings despite the difficulties along the way [58]. In this case, 560 contacts successfully completed the contact tracing program during 2020 and 2021, which likely contributed to preventing SARS-CoV-2 infections. Nevertheless, implementing quality improvement cycles from the outset (challenging due to the overwhelming workload of CES staff at the time) would have been helpful in identifying and mitigating factors that hindered implementation and affected intervention outcomes.

### Limitations of the analysis

An important limitation of this analysis is that it relies on pre-existing programmatic data sources, which can include inaccurate or missing data. As described above, technical challenges related to digital data collection likely resulted in some home visits being unrecorded, which would result in an underestimation of the performance of our contact tracing program. Inconsistencies in dates of visits were common, likely due to human error when entering these data into the App, leading to an inability to assess the time between a contact’s identification and their first and subsequent follow-up visits, which would have been informative indicators of compliance with contact tracing guidelines. Making the data collection date field in the App more intuitive and making both staff at the clinic and CHWs aware of the importance of correctly completing these fields will be key to assessing the timeliness of the intervention in the future and to identify significant delays in contact tracing that may affect the effectiveness of the intervention [59]. Similarly, the high levels of missingness for some variables, such as comorbidities or whether a contact lived with a case, affects the accuracy of our results. We also found differences in the prevalence of diagnosed diabetes, diagnosed hypertension, and tobacco use between the contacts interviewed in the study and the Chiapas state estimates from national surveys (5.15% and 7.80% [40], 3.86% and 16.20% [40], and 1.34% and 7.60% [41], respectively) potentially indicating underreporting in the self-reported disease statuses. This issue could be addressed by making fields related to these variables mandatory in the contact tracing App. Following reference guidelines [60], in order to identify and correct incomplete and inconsistent data recording in time, data quality assessment and improvement should be performed more frequently.

## Conclusions

Despite the limitations of this study and the poorer performance of our CHW-led contact tracing intervention relative to other contact tracing interventions, to our knowledge, our study is the first data-driven evaluation in LAC of a CHW-led COVID-19 contact tracing intervention. Our in-depth, data-based assessment of implementation challenges underscores the importance of early and ongoing evaluation of these programs to detect pitfalls that may be limiting the effectiveness of interventions, which is particularly relevant when resources are limited, as they can inform more optimal use of existing resources.

### Supplementary Information

**Additional file 1. **The Partners In Health Cross-Site COVID-19 Cohort Technical Working Group.

## Data Availability

The data presented in this study are available on request from the corresponding author. The data are not publicly available due to privacy and confidentiality.

## Abbreviations

App: Application
CES: Compañeros En Salud
CHW: Community Health Worker
LAC: Latin America and the Caribbean
MXN: Mexican Pesos
MoH: Ministry of Health
PPE: Personal Protective Equipment
USD: United States Dollar
