# Supplementary material for: Evaluation of the implementation of a community health worker-led COVID-19 contact tracing intervention in Chiapas, Mexico, from March 2020 to December 2021
Source: BMC Health Serv Res. 2024 Jan 18;24:97. doi: 10.1186/s12913-024-10590-3 (PMC10795220; doi:10.1186/s12913-024-10590-3)
Supplement: Supplementary file 1 — Additional file 1. The Partners In Health Cross-Site COVID-19 Cohort Technical Working Group. [file 12913_2024_10590_MOESM1_ESM.docx]

**The Partners In Health Cross-Site COVID-19 Cohort Technical Working Group**

Jean Claude Mugunga^1^, Donald Fejfar^1^, Stefanie Joseph^1^, Wesler Lambert^2^, Mary Clisbee^2^, Fernet Leandre^2^, Prince F. Varney^3^, Melino Ndayizigiye^4^, Patrick Nkundanyirazo^4^, Afom Andom^4^, Emilia Connolly^5^, Chiyembekezo Kachimanga^5^, Fabien Munyaneza^5^, Zeus Aranda^6,7^, Jesus Peinado^8^, Marco Tovar^8^, Vincent Cubaka^9^, Nadine Karema^9^, Foday Boima^10^, Gregory Jerome^10^, Bethany Hedt-Gauthier^11^, Isabel R. Fulcher^11,12^, Dale A. Barnhart^9,11^, Megan Murray^11^

^1^Partners In Health, Boston, MA, USA. ^2^Partners In Health Haiti (Zanmi Lasante), Croix-des-Bouquets, Haiti. ^3^Partners In Health Liberia, Harper, Liberia. ^4^Partners In Health Lesotho, Maseru, Lesotho. ^5^Partners In Health Malawi (Abwenzi Pa Za Umoyo), Neno, Malawi. ^6^Partners In Health Mexico (Compañeros En Salud), Ángel Albino Corzo, México. ^7^Departamento de Salud, El Colegio de la Frontera Sur, San Cristóbal de las Casas, México. ^8^Partners In Health Peru (Socios En Salud), Lima, Perú. ^9^Partners In Health Rwanda (Inshuti Mu Buzima), Kigali, Rwanda. ^10^Partners In Health Sierra Leone, Koidu, Sierra Leone. ^11^Department of Global Health and Social Medicine, Harvard Medical School, Boston, MA, USA. ^12^Harvard Data Science Initiative, Boston, MA, USA.
